# 6β-Methyl-3,20-dioxopregn-4-en-17-yl acetate

**DOI:** 10.1107/S1600536812008665

**Published:** 2012-03-10

**Authors:** Longran Chen, Xuefen Liu, Peng Yang

**Affiliations:** a Qianjiang Colloge, Hangzhou Normal University, Hangzhou 310012, People’s Republic of China

## Abstract

The title compound, C_24_H_34_O_4_, is a precursor of Megestrol acetate. Ring *A* has a half-chair conformation [*Q* = 0.446 (3) Å, θ = 54.6 (4)° and ϕ = 9.5 (4)°]. Ring *D* adopts a 13β-envelope conformation [*Q* = 0.463 (2) Å and ϕ = 188.2 (3)°].

## Related literature

For the characterization of related structures, see: Evans & Boeyens (1989 ▶). Soriano-Garcia *et al.* (2005 ▶). Yousuf *et al.* (2011 ▶). For the physiological properties of the title compound, see: Mishell (1996 ▶).
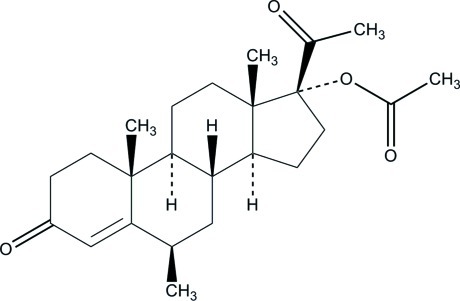


## Experimental

### 

#### Crystal data

C_24_H_34_O_4_
*M* *_r_* = 386.51
Orthorhombic, 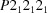

*a* = 10.0411 (3) Å
*b* = 11.3123 (3) Å
*c* = 18.5549 (7) Å
*V* = 2107.61 (12) Å^3^
*Z* = 4
Mo *K*α radiation
μ = 0.08 mm^−1^
*T* = 296 K
0.56 × 0.52 × 0.31 mm

#### Data collection

Rigaku R-AXIS RAPID/ZJUG diffractometer
Absorption correction: multi-scan (*ABSCOR*; Higashi, 1995 ▶) *T*_min_ = 0.946, *T*_max_ = 0.975
20014 measured reflections
2679 independent reflections
2108 reflections with *I* > 2σ(*I*)
*R*_int_ = 0.035

#### Refinement

*R*[*F* ^2^ > 2σ(*F* ^2^)] = 0.039
*wR*(*F* ^2^) = 0.102
*S* = 1.00
2679 reflections
259 parameters
H-atom parameters constrained
Δρ_max_ = 0.17 e Å^−3^
Δρ_min_ = −0.16 e Å^−3^

### 

Data collection: *PROCESS-AUTO* (Rigaku, 2006 ▶); cell refinement: *PROCESS-AUTO*; data reduction: *CrystalStructure* (Rigaku, 2007 ▶); program(s) used to solve structure: *SHELXS97* (Sheldrick, 2008 ▶); program(s) used to refine structure: *SHELXL97* (Sheldrick, 2008 ▶); molecular graphics: *ORTEP-3 for Windows* (Farrugia, 1997 ▶); software used to prepare material for publication: *WinGX* (Farrugia, 1999 ▶).

## Supplementary Material

Crystal structure: contains datablock(s) global, I. DOI: 10.1107/S1600536812008665/ld2047sup1.cif

Structure factors: contains datablock(s) I. DOI: 10.1107/S1600536812008665/ld2047Isup2.hkl

Additional supplementary materials:  crystallographic information; 3D view; checkCIF report

## Figures and Tables

**Fig. 1.**
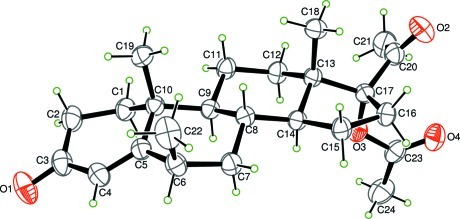
The molecular structure of (I) with displacement ellipsoids drawn at the 30% probability level.

## supplementary crystallographic information

### Comment

6β-methyl-3,20-dioxopregn-4-ene-17-yl acetate and its derivatives (for example, Megestrol acetate) are important progestines used for the treatment of endomentriosis as well as several other applications (Mishell, 1996).

The structure of the title compound is depicted in Fig.1. Ring A (C1—C5, C10) has a slightly distorted half-chair conformation with Q (total puckering amplitude) = 0.446 (3) Å, theta (azimuthal angle) = 54.6 (4) °, φ (phase angle) = 9.5 (4) °, while ring B (C5—C10) and C (C8,C9, C11—C14) are in chair conformation [ring B: Q = 0.521 (2) Å, theta = 12.7 (2) °, φ = 188.7 (13) °; ring C: Q = 0.564 (2) Å, theta = 2.8 (2) °, φ = 267 (4) °] (Evans & Boeyens, 1989). The five-membered ring D exhibits a 13β-envelope conformation with puckering amplitude Q = 0.463 (2) Å and phase angle= 188.2 (3) °. The crystals are isomorphous to 17alpha-Acetoxy-6-methylene-4-pregnene-3,20-dione (Soriano-Garcia *et al.*, 2005). The crystal structure is also similar to 3alpha-Dimethylamino-20-(*N*-methylacetamido)pregn-5-ene (Yousuf *et al.*, 2011).

### Experimental

The title compound was received from Shanghai Xinhualian Pharmaceutical Co., Ltd. Suitable crystals were obtained by slow evaporation of its ethanol solution at room temperature.

### Refinement

All H atoms were initially located in a difference Fourier map. The methyl H atoms were then constrained to an ideal geometry with C—H distances of 0.98 Å and *U*_iso_(H) = 1.5*U*_eq_(C), but each group was allowed to rotate freely about its C—C bond. All other H atoms were placed in geometrically idealized positions and constrained to ride on their parent atoms with C—H distances in the range 0.93–0.98 Å and *U*_iso_(H) = 1.2*U*_eq_(C).

### Crystal data

**Table:**

C_24_H_34_O_4_	*F*(000) = 840
*M**_r_* = 386.51	*D*_x_ = 1.218 Mg m^−^^3^
Orthorhombic, *P*2_1_2_1_2_1_	Mo *K*α radiation, λ = 0.71073 Å
Hall symbol: P 2ac 2ab	Cell parameters from 14918 reflections
*a* = 10.0411 (3) Å	θ = 3.0–27.4°
*b* = 11.3123 (3) Å	µ = 0.08 mm^−^^1^
*c* = 18.5549 (7) Å	*T* = 296 K
*V* = 2107.61 (12) Å^3^	Chunk, colorless
*Z* = 4	0.56 × 0.52 × 0.31 mm

### Data collection

**Table:**

Rigaku R-AXIS RAPID/ZJUG diffractometer	2679 independent reflections
Radiation source: rolling anode	2108 reflections with *I* > 2σ(*I*)
Graphite monochromator	*R*_int_ = 0.035
Detector resolution: 10.00 pixels mm^-1^	θ_max_ = 27.4°, θ_min_ = 3.0°
ω scans	*h* = −12→11
Absorption correction: multi-scan (*ABSCOR*; Higashi, 1995)	*k* = −14→14
*T*_min_ = 0.946, *T*_max_ = 0.975	*l* = −24→24
20014 measured reflections	

### Refinement

**Table:**

Refinement on *F*^2^	Secondary atom site location: difference Fourier map
Least-squares matrix: full	Hydrogen site location: inferred from neighbouring sites
*R*[*F*^2^ > 2σ(*F*^2^)] = 0.039	H-atom parameters constrained
*wR*(*F*^2^) = 0.102	*w* = 1/[σ^2^(*F*_o_^2^) + (0.0441*P*)^2^ + 0.5695*P*] where *P* = (*F*_o_^2^ + 2*F*_c_^2^)/3
*S* = 1.00	(Δ/σ)_max_ = 0.001
2679 reflections	Δρ_max_ = 0.17 e Å^−^^3^
259 parameters	Δρ_min_ = −0.16 e Å^−^^3^
0 restraints	Extinction correction: *SHELXL97* (Sheldrick, 2008), Fc^*^=kFc[1+0.001xFc^2^λ^3^/sin(2θ)]^-1/4^
Primary atom site location: structure-invariant direct methods	Extinction coefficient: 0.0057 (12)

### Special details

**Table:**

Geometry. All e.s.d.'s (except the e.s.d. in the dihedral angle between two l.s. planes) are estimated using the full covariance matrix. The cell e.s.d.'s are taken into account individually in the estimation of e.s.d.'s in distances, angles and torsion angles; correlations between e.s.d.'s in cell parameters are only used when they are defined by crystal symmetry. An approximate (isotropic) treatment of cell e.s.d.'s is used for estimating e.s.d.'s involving l.s. planes.
Refinement. Refinement of *F*^2^ against ALL reflections. The weighted *R*-factor *wR* and goodness of fit *S* are based on *F*^2^, conventional *R*-factors *R* are based on *F*, with *F* set to zero for negative *F*^2^. The threshold expression of *F*^2^ > σ(*F*^2^) is used only for calculating *R*-factors(gt) *etc*. and is not relevant to the choice of reflections for refinement. *R*-factors based on *F*^2^ are statistically about twice as large as those based on *F*, and *R*- factors based on ALL data will be even larger.

### Fractional atomic coordinates and isotropic or equivalent isotropic displacement parameters (Å^2^)

**Table:**

	*x*	*y*	*z*	*U*_iso_*/*U*_eq_	
O1	0.9284 (2)	−0.06142 (15)	0.53897 (13)	0.0759 (7)	
O2	0.6725 (3)	0.92350 (17)	0.70081 (12)	0.0713 (6)	
O3	0.49550 (16)	0.66587 (14)	0.65423 (9)	0.0439 (4)	
O4	0.3669 (2)	0.82806 (18)	0.65415 (12)	0.0655 (6)	
C1	0.8845 (3)	0.1906 (2)	0.65614 (14)	0.0475 (6)	
H1A	0.9083	0.2115	0.7052	0.057*	
H1B	0.7889	0.1785	0.6547	0.057*	
C2	0.9537 (3)	0.0745 (2)	0.63640 (16)	0.0572 (7)	
H2A	1.0487	0.0825	0.6445	0.069*	
H2B	0.9210	0.0120	0.6676	0.069*	
C3	0.9299 (3)	0.0410 (2)	0.55974 (17)	0.0533 (7)	
C4	0.9105 (3)	0.1385 (2)	0.50972 (15)	0.0499 (6)	
H4	0.8976	0.1195	0.4615	0.060*	
C5	0.9097 (2)	0.2534 (2)	0.52737 (13)	0.0407 (5)	
C6	0.8994 (3)	0.3438 (2)	0.46795 (14)	0.0461 (6)	
H6	0.8611	0.3028	0.4263	0.055*	
C7	0.8027 (3)	0.4433 (2)	0.48694 (13)	0.0447 (6)	
H7A	0.8107	0.5055	0.4512	0.054*	
H7B	0.7124	0.4130	0.4848	0.054*	
C8	0.8270 (2)	0.49588 (19)	0.56153 (12)	0.0368 (5)	
H8	0.9157	0.5320	0.5629	0.044*	
C9	0.8186 (2)	0.39747 (19)	0.61904 (12)	0.0373 (5)	
H9	0.7299	0.3622	0.6139	0.045*	
C10	0.9205 (2)	0.29462 (19)	0.60597 (13)	0.0388 (5)	
C11	0.8245 (3)	0.4476 (2)	0.69591 (12)	0.0441 (6)	
H11A	0.8099	0.3838	0.7299	0.053*	
H11B	0.9129	0.4791	0.7045	0.053*	
C12	0.7216 (3)	0.5454 (2)	0.71000 (13)	0.0443 (6)	
H12A	0.6327	0.5120	0.7083	0.053*	
H12B	0.7354	0.5779	0.7578	0.053*	
C13	0.7333 (2)	0.64437 (19)	0.65394 (13)	0.0362 (5)	
C14	0.7224 (2)	0.58932 (19)	0.57819 (12)	0.0360 (5)	
H14	0.6363	0.5487	0.5768	0.043*	
C15	0.7092 (3)	0.69619 (19)	0.52808 (13)	0.0436 (6)	
H15A	0.6611	0.6753	0.4846	0.052*	
H15B	0.7961	0.7267	0.5149	0.052*	
C16	0.6308 (3)	0.7875 (2)	0.57264 (13)	0.0441 (6)	
H16A	0.5437	0.8007	0.5515	0.053*	
H16B	0.6782	0.8622	0.5740	0.053*	
C17	0.6167 (2)	0.7366 (2)	0.64927 (13)	0.0393 (5)	
C22	1.0348 (3)	0.3910 (3)	0.44369 (17)	0.0646 (8)	
H18A	1.0727	0.4386	0.4814	0.097*	
H18B	1.0237	0.4383	0.4011	0.097*	
H18C	1.0931	0.3260	0.4334	0.097*	
C19	1.0656 (2)	0.3338 (2)	0.62124 (16)	0.0507 (6)	
H19A	1.0760	0.3491	0.6718	0.076*	
H19B	1.0848	0.4045	0.5945	0.076*	
H19C	1.1257	0.2723	0.6068	0.076*	
C18	0.8651 (3)	0.7121 (2)	0.66410 (15)	0.0493 (6)	
H20A	0.8674	0.7463	0.7114	0.074*	
H20B	0.8715	0.7735	0.6286	0.074*	
H20C	0.9386	0.6585	0.6586	0.074*	
C20	0.6198 (3)	0.8284 (2)	0.70979 (15)	0.0507 (6)	
C21	0.5662 (4)	0.7928 (3)	0.78251 (15)	0.0689 (9)	
H22A	0.6221	0.8248	0.8197	0.103*	
H22B	0.5651	0.7081	0.7861	0.103*	
H22C	0.4773	0.8227	0.7880	0.103*	
C23	0.3771 (3)	0.7220 (3)	0.65162 (15)	0.0502 (6)	
C24	0.2663 (3)	0.6356 (3)	0.6461 (2)	0.0717 (9)	
H24A	0.1828	0.6766	0.6486	0.108*	
H24B	0.2722	0.5800	0.6851	0.108*	
H24C	0.2724	0.5943	0.6010	0.108*	

### Atomic displacement parameters (Å^2^)

**Table:**

	*U*^11^	*U*^22^	*U*^33^	*U*^12^	*U*^13^	*U*^23^
O1	0.0979 (17)	0.0353 (10)	0.0944 (16)	−0.0064 (10)	0.0245 (14)	−0.0033 (10)
O2	0.0876 (16)	0.0496 (11)	0.0766 (14)	−0.0048 (11)	0.0016 (13)	−0.0149 (10)
O3	0.0355 (9)	0.0459 (9)	0.0503 (9)	0.0016 (7)	0.0036 (8)	0.0031 (8)
O4	0.0601 (12)	0.0629 (12)	0.0734 (13)	0.0205 (10)	0.0049 (12)	0.0007 (11)
C1	0.0506 (14)	0.0424 (12)	0.0495 (14)	0.0014 (11)	0.0012 (13)	0.0087 (11)
C2	0.0604 (17)	0.0420 (13)	0.0692 (19)	0.0045 (12)	0.0028 (14)	0.0098 (13)
C3	0.0484 (15)	0.0385 (13)	0.0729 (19)	−0.0019 (11)	0.0133 (14)	0.0025 (13)
C4	0.0523 (16)	0.0423 (13)	0.0552 (15)	−0.0034 (11)	0.0058 (13)	−0.0045 (11)
C5	0.0352 (13)	0.0393 (11)	0.0474 (13)	−0.0006 (10)	0.0043 (11)	0.0000 (10)
C6	0.0541 (15)	0.0419 (12)	0.0424 (13)	0.0001 (11)	0.0059 (12)	−0.0013 (10)
C7	0.0543 (15)	0.0425 (12)	0.0373 (12)	0.0042 (12)	0.0007 (11)	0.0025 (10)
C8	0.0384 (12)	0.0354 (11)	0.0365 (12)	−0.0014 (9)	0.0027 (10)	0.0020 (9)
C9	0.0364 (12)	0.0365 (11)	0.0389 (12)	−0.0004 (9)	0.0011 (10)	0.0024 (9)
C10	0.0379 (13)	0.0354 (11)	0.0432 (13)	−0.0005 (10)	0.0006 (10)	0.0024 (10)
C11	0.0502 (14)	0.0445 (13)	0.0377 (12)	0.0076 (11)	0.0003 (11)	0.0037 (10)
C12	0.0511 (15)	0.0468 (13)	0.0350 (12)	0.0062 (11)	0.0044 (11)	0.0055 (10)
C13	0.0370 (12)	0.0376 (11)	0.0341 (11)	0.0020 (9)	0.0009 (10)	0.0020 (9)
C14	0.0389 (12)	0.0350 (11)	0.0341 (12)	−0.0006 (9)	−0.0001 (10)	0.0038 (9)
C15	0.0525 (14)	0.0392 (12)	0.0390 (12)	0.0024 (11)	0.0031 (11)	0.0067 (10)
C16	0.0476 (14)	0.0396 (12)	0.0452 (13)	0.0045 (11)	0.0028 (11)	0.0075 (10)
C17	0.0389 (12)	0.0382 (11)	0.0407 (12)	−0.0004 (10)	0.0004 (11)	−0.0006 (10)
C22	0.070 (2)	0.0561 (16)	0.0677 (19)	0.0004 (14)	0.0255 (16)	0.0074 (15)
C19	0.0370 (13)	0.0499 (14)	0.0651 (17)	0.0009 (11)	−0.0039 (12)	−0.0018 (13)
C18	0.0437 (14)	0.0479 (13)	0.0563 (16)	−0.0037 (11)	−0.0046 (12)	−0.0065 (12)
C20	0.0540 (15)	0.0480 (14)	0.0502 (15)	0.0101 (13)	−0.0013 (13)	−0.0058 (12)
C21	0.087 (2)	0.074 (2)	0.0448 (16)	0.0202 (18)	0.0032 (16)	−0.0082 (14)
C23	0.0421 (14)	0.0628 (17)	0.0457 (14)	0.0108 (12)	0.0045 (12)	0.0053 (13)
C24	0.0409 (15)	0.088 (2)	0.086 (2)	−0.0019 (15)	0.0033 (16)	0.0111 (19)

### Geometric parameters (Å, º)

**Table:**

O1—C3	1.221 (3)	C12—C13	1.533 (3)
O2—C20	1.210 (3)	C12—H12A	0.9700
O3—C23	1.348 (3)	C12—H12B	0.9700
O3—C17	1.459 (3)	C13—C18	1.540 (3)
O4—C23	1.205 (3)	C13—C14	1.541 (3)
C1—C2	1.530 (3)	C13—C17	1.571 (3)
C1—C10	1.544 (3)	C14—C15	1.531 (3)
C1—H1A	0.9700	C14—H14	0.9800
C1—H1B	0.9700	C15—C16	1.539 (3)
C2—C3	1.491 (4)	C15—H15A	0.9700
C2—H2A	0.9700	C15—H15B	0.9700
C2—H2B	0.9700	C16—C17	1.540 (3)
C3—C4	1.455 (4)	C16—H16A	0.9700
C4—C5	1.341 (3)	C16—H16B	0.9700
C4—H4	0.9300	C17—C20	1.530 (3)
C5—C6	1.508 (3)	C22—H18A	0.9600
C5—C10	1.535 (3)	C22—H18B	0.9600
C6—C7	1.527 (3)	C22—H18C	0.9600
C6—C22	1.529 (4)	C19—H19A	0.9600
C6—H6	0.9800	C19—H19B	0.9600
C7—C8	1.526 (3)	C19—H19C	0.9600
C7—H7A	0.9700	C18—H20A	0.9600
C7—H7B	0.9700	C18—H20B	0.9600
C8—C14	1.522 (3)	C18—H20C	0.9600
C8—C9	1.544 (3)	C20—C21	1.508 (4)
C8—H8	0.9800	C21—H22A	0.9600
C9—C11	1.536 (3)	C21—H22B	0.9600
C9—C10	1.568 (3)	C21—H22C	0.9600
C9—H9	0.9800	C23—C24	1.485 (4)
C10—C19	1.549 (3)	C24—H24A	0.9600
C11—C12	1.536 (3)	C24—H24B	0.9600
C11—H11A	0.9700	C24—H24C	0.9600
C11—H11B	0.9700		
			
C23—O3—C17	118.33 (17)	C12—C13—C14	108.56 (18)
C2—C1—C10	113.8 (2)	C18—C13—C14	111.9 (2)
C2—C1—H1A	108.8	C12—C13—C17	117.73 (19)
C10—C1—H1A	108.8	C18—C13—C17	108.48 (18)
C2—C1—H1B	108.8	C14—C13—C17	99.50 (18)
C10—C1—H1B	108.8	C8—C14—C15	119.02 (19)
H1A—C1—H1B	107.7	C8—C14—C13	114.65 (19)
C3—C2—C1	112.0 (2)	C15—C14—C13	103.95 (17)
C3—C2—H2A	109.2	C8—C14—H14	106.1
C1—C2—H2A	109.2	C15—C14—H14	106.1
C3—C2—H2B	109.2	C13—C14—H14	106.1
C1—C2—H2B	109.2	C14—C15—C16	104.36 (18)
H2A—C2—H2B	107.9	C14—C15—H15A	110.9
O1—C3—C4	121.1 (3)	C16—C15—H15A	110.9
O1—C3—C2	123.0 (3)	C14—C15—H15B	110.9
C4—C3—C2	115.9 (2)	C16—C15—H15B	110.9
C5—C4—C3	125.5 (3)	H15A—C15—H15B	108.9
C5—C4—H4	117.3	C15—C16—C17	106.98 (18)
C3—C4—H4	117.3	C15—C16—H16A	110.3
C4—C5—C6	118.7 (2)	C17—C16—H16A	110.3
C4—C5—C10	121.7 (2)	C15—C16—H16B	110.3
C6—C5—C10	119.59 (19)	C17—C16—H16B	110.3
C5—C6—C7	112.0 (2)	H16A—C16—H16B	108.6
C5—C6—C22	113.0 (2)	O3—C17—C20	110.0 (2)
C7—C6—C22	112.1 (2)	O3—C17—C16	109.87 (19)
C5—C6—H6	106.4	C20—C17—C16	114.95 (19)
C7—C6—H6	106.4	O3—C17—C13	104.72 (16)
C22—C6—H6	106.4	C20—C17—C13	113.3 (2)
C8—C7—C6	113.3 (2)	C16—C17—C13	103.33 (19)
C8—C7—H7A	108.9	C6—C22—H18A	109.5
C6—C7—H7A	108.9	C6—C22—H18B	109.5
C8—C7—H7B	108.9	H18A—C22—H18B	109.5
C6—C7—H7B	108.9	C6—C22—H18C	109.5
H7A—C7—H7B	107.7	H18A—C22—H18C	109.5
C14—C8—C7	110.18 (19)	H18B—C22—H18C	109.5
C14—C8—C9	108.82 (18)	C10—C19—H19A	109.5
C7—C8—C9	109.70 (18)	C10—C19—H19B	109.5
C14—C8—H8	109.4	H19A—C19—H19B	109.5
C7—C8—H8	109.4	C10—C19—H19C	109.5
C9—C8—H8	109.4	H19A—C19—H19C	109.5
C11—C9—C8	111.91 (18)	H19B—C19—H19C	109.5
C11—C9—C10	113.11 (19)	C13—C18—H20A	109.5
C8—C9—C10	113.10 (18)	C13—C18—H20B	109.5
C11—C9—H9	106.0	H20A—C18—H20B	109.5
C8—C9—H9	106.0	C13—C18—H20C	109.5
C10—C9—H9	106.0	H20A—C18—H20C	109.5
C5—C10—C1	108.96 (19)	H20B—C18—H20C	109.5
C5—C10—C19	109.1 (2)	O2—C20—C21	121.1 (3)
C1—C10—C19	109.2 (2)	O2—C20—C17	120.7 (2)
C5—C10—C9	109.04 (19)	C21—C20—C17	117.9 (2)
C1—C10—C9	108.65 (19)	C20—C21—H22A	109.5
C19—C10—C9	111.90 (19)	C20—C21—H22B	109.5
C12—C11—C9	113.5 (2)	H22A—C21—H22B	109.5
C12—C11—H11A	108.9	C20—C21—H22C	109.5
C9—C11—H11A	108.9	H22A—C21—H22C	109.5
C12—C11—H11B	108.9	H22B—C21—H22C	109.5
C9—C11—H11B	108.9	O4—C23—O3	122.9 (3)
H11A—C11—H11B	107.7	O4—C23—C24	126.4 (3)
C13—C12—C11	111.03 (19)	O3—C23—C24	110.7 (2)
C13—C12—H12A	109.4	C23—C24—H24A	109.5
C11—C12—H12A	109.4	C23—C24—H24B	109.5
C13—C12—H12B	109.4	H24A—C24—H24B	109.5
C11—C12—H12B	109.4	C23—C24—H24C	109.5
H12A—C12—H12B	108.0	H24A—C24—H24C	109.5
C12—C13—C18	110.2 (2)	H24B—C24—H24C	109.5
			
C10—C1—C2—C3	−54.3 (3)	C11—C12—C13—C14	54.6 (3)
C1—C2—C3—O1	−151.0 (3)	C11—C12—C13—C17	166.5 (2)
C1—C2—C3—C4	30.0 (3)	C7—C8—C14—C15	−58.9 (3)
O1—C3—C4—C5	179.6 (3)	C9—C8—C14—C15	−179.2 (2)
C2—C3—C4—C5	−1.4 (4)	C7—C8—C14—C13	177.23 (19)
C3—C4—C5—C6	175.1 (2)	C9—C8—C14—C13	56.9 (2)
C3—C4—C5—C10	−4.2 (4)	C12—C13—C14—C8	−58.6 (2)
C4—C5—C6—C7	137.0 (3)	C18—C13—C14—C8	63.3 (2)
C10—C5—C6—C7	−43.6 (3)	C17—C13—C14—C8	177.79 (18)
C4—C5—C6—C22	−95.2 (3)	C12—C13—C14—C15	169.82 (19)
C10—C5—C6—C22	84.1 (3)	C18—C13—C14—C15	−68.3 (2)
C5—C6—C7—C8	49.1 (3)	C17—C13—C14—C15	46.2 (2)
C22—C6—C7—C8	−79.2 (3)	C8—C14—C15—C16	−162.4 (2)
C6—C7—C8—C14	−176.85 (19)	C13—C14—C15—C16	−33.4 (2)
C6—C7—C8—C9	−57.1 (3)	C14—C15—C16—C17	6.7 (3)
C14—C8—C9—C11	−52.1 (3)	C23—O3—C17—C20	58.0 (3)
C7—C8—C9—C11	−172.7 (2)	C23—O3—C17—C16	−69.5 (2)
C14—C8—C9—C10	178.72 (19)	C23—O3—C17—C13	−179.9 (2)
C7—C8—C9—C10	58.1 (3)	C15—C16—C17—O3	−89.6 (2)
C4—C5—C10—C1	−18.7 (3)	C15—C16—C17—C20	145.7 (2)
C6—C5—C10—C1	161.9 (2)	C15—C16—C17—C13	21.8 (2)
C4—C5—C10—C19	100.4 (3)	C12—C13—C17—O3	−43.0 (3)
C6—C5—C10—C19	−79.0 (3)	C18—C13—C17—O3	−169.06 (19)
C4—C5—C10—C9	−137.2 (2)	C14—C13—C17—O3	73.86 (19)
C6—C5—C10—C9	43.5 (3)	C12—C13—C17—C20	76.9 (3)
C2—C1—C10—C5	47.2 (3)	C18—C13—C17—C20	−49.1 (3)
C2—C1—C10—C19	−71.8 (3)	C14—C13—C17—C20	−166.2 (2)
C2—C1—C10—C9	165.9 (2)	C12—C13—C17—C16	−158.1 (2)
C11—C9—C10—C5	−178.3 (2)	C18—C13—C17—C16	75.9 (2)
C8—C9—C10—C5	−49.8 (2)	C14—C13—C17—C16	−41.2 (2)
C11—C9—C10—C1	63.1 (3)	O3—C17—C20—O2	−148.1 (3)
C8—C9—C10—C1	−168.4 (2)	C16—C17—C20—O2	−23.4 (4)
C11—C9—C10—C19	−57.5 (3)	C13—C17—C20—O2	95.1 (3)
C8—C9—C10—C19	71.0 (3)	O3—C17—C20—C21	37.2 (3)
C8—C9—C11—C12	52.9 (3)	C16—C17—C20—C21	161.9 (3)
C10—C9—C11—C12	−177.95 (19)	C13—C17—C20—C21	−79.6 (3)
C9—C11—C12—C13	−54.2 (3)	C17—O3—C23—O4	−9.2 (4)
C11—C12—C13—C18	−68.4 (2)	C17—O3—C23—C24	171.3 (2)

## References

[bb1] Evans, D. G. & Boeyens, J. C. A. (1989). *Acta Cryst.* B**45**, 581–590.

[bb2] Farrugia, L. J. (1997). *J. Appl. Cryst.* **30**, 565.

[bb3] Farrugia, L. J. (1999). *J. Appl. Cryst.* **32**, 837–838.

[bb4] Higashi, T. (1995). *ABSCOR* Rigaku Corporation, Tokyo, Japan.

[bb5] Mishell, D. R. Jr (1996). *J. Reprod. Med.* **41**, 381–390. 8725700

[bb6] Rigaku (2006). *PROCESS-AUTO* Rigaku Corporation,Tokyo, Japan.

[bb7] Rigaku (2007). *CrystalStructure* Rigaku Corporation, Tokyo, Japan.

[bb8] Sheldrick, G. M. (2008). *Acta Cryst.* A**64**, 112–122. 10.1107/S0108767307043930 18156677

[bb9] Soriano-Garcia, M., Flores, E., Bratoeff, E., Ramirez, E., Cabeza, M. & Rodriguez, J. G. A. (2005). *Anal Sci. X-Ray Struct Anal Online*, **21**, x27–x28.

[bb10] Yousuf, S., Musharraf, S. G., Iqbal, N., Adhikari, A. & Choudhary, M. I. (2011). *Acta Cryst.* E**67**, o2918. 10.1107/S160053681103964X PMC3247332 22219950

